# Supplementary material for: Differential BACH1 Expression in Basal-like Breast Tumors of Black Women Identified via Immunohistochemistry
Source: Curr Oncol. 2025 Jul 14;32(7):404. doi: 10.3390/curroncol32070404 (PMC12293708; doi:10.3390/curroncol32070404)
Supplement: Supplementary file 1 [file curroncol-32-00404-s001.zip › curroncol-3642447-supplementary.pdf]

N.M.Dowling et al.
Supplementary Data Table S1

Group Descriptive – Tumor Size and BACH1 IHC score

Tumor size group (diameter)	N	Mean	SD	SE	Coefficient of variation
3-25 mm	65	3.292	1.693	0.210	0.514
27-85 mm	50	4.200	1.796	0.254	0.428

Supplementary Data Table S1. BACH1 IHC images and group description based on tumor size in diameter
Group description based on tumor sizes with BACH1 scores. The group (N = 50) with larger tumor sizes (27-85 mm in diameter) has a higher mean BACH1 scores than the other (N = 65) with smaller tumor sizes (3-25 mm in diameter).

N.M.Dowling et al.
Supplementary Data Figure S1

A

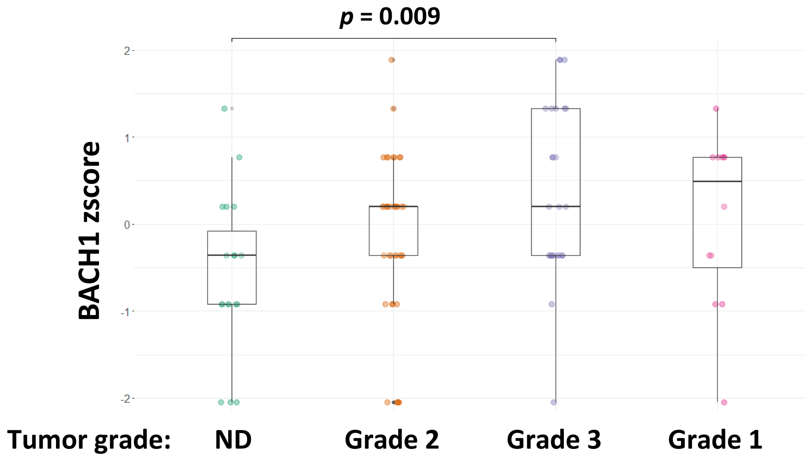

BACH1 IHC Zscore	Grade I	Grade II	Grade III	ND	Total
Median	0.485	0.204	0.204	-0.358	0.204
Mean	0.064	0.377	-0.133	-0.566	0.000
N	12	26	40	19	97

B

Two Group Comparison		P unadjusted	P adjusted
ND	Grade 2	0.0618	0.2474
**ND**	**Grade 3**	**0.0015**	**0.0092**
Grade 2	Grade 3	0.0840	0.2521
**Grade 3**	**Grade I**	**0.0488**	0.2444
Grade 2	Grade I	0.5314	0.5314
ND	Grade I	0.5112	1.0000

Supplementary Data Figure S1. BACH1 expression is higher in the tumor grade 3 than grade 1 or unclassified group (ND)
(A) Box plots indicating BACH1 IHC zscores by breast cancer grades. Median and mean of BACH1 IHC zscore in each groups, Grade 1 (N = 23), Grade 2 (N = 40), Grade 3 (N = 26), and Not Determined (N = 19), are shown. (**B**) Kruskal-Wallis multiple comparison with p-values adjusted with the Holm method are shown in the table.

N.M.Dowling et al.
Supplementary Data Figure S2

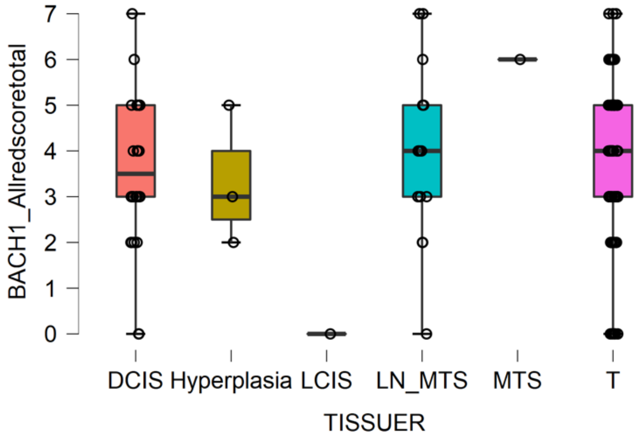

BACH1 IHC scores

Tissue	DCIS	Hyperplasia	LCIS	LN_MTS	MTS	T
**N**	28	3	1	14	1	77
**Min**	0	2	0	0	6	0
**Max**	7	5	0	7	6	7

Supplementary Data Figure S2. BACH1 expression analysis using IHC scores among breast tumor tissue types

BACH1 expression levels by IHC scores are similar in tumor types in a boxplot. Total numbers of patient tissues with maximum and minimum BACH1 IHC scores (Allredscoretotal) are shown by tissue types in the table. (DCIS: ductal carcinoma *in situ*, LCIS: lobular carcinoma *in situ*, LN-MTS: lymph node metastasis, MTS: metastasis, T: tumor). Metastases (MTS) and LCIS (luminal carcinoma *in situ*) were excluded in this comparison due to the limited sample size (N = 1 per group).

A

	Subgroups	Number (N)	Mean Rank	Sum of Ranks
**BACH1 Zscore (Allredscoretotal)**	**Non-invasive (Non-metastatic)**	109	61.48	6701.50
	**Invasive (Metastatic)**	15	69.90	1048.50
	**Total**	124		

Test Statistics (Mann-Whitney U and Wilcoxon W tests)	
	Zscore: BACH1_Allredscoretotal
Mann-Whitney U	706.500
Wilcoxon W	6701.500
Z	-.866
Asymp. Sig. (2-tailed)	.387
Exact Sig. (2-tailed)	.392
Exact Sig. (1-tailed)	.196
Point Probability	.001
Grouping Variable: Invasive vs Non-Invasive Cancer	

B

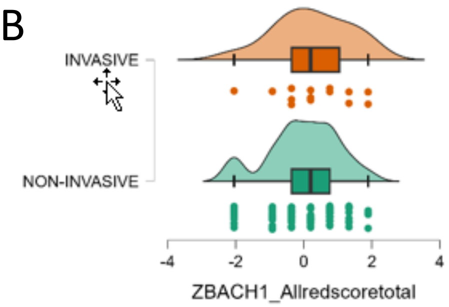

Supplementary Data Figure S3. BACH1 expression analysis using IHC scores by tumor invasiveness (A) Tables indicating tumors are divided by two subgroups, invasive vs. non-invasive (equivalent to metastatic vs. non-metastatic). Invasive group (metastatic group, N = 15) contains cancer types of MTS and LN_MTS, whereas non-invasive group (non-metastatic group, N = 109) contains cancer types of T, DCIS, and hyperplasia for BACH1 IHC analysis using Mann-Whitney and Wilcoxon W tests and the test results are shown. (B) Distribution plot of BACH1 IHC scores by subgroups; invasive (metastatic) vs. non-invasive (non-metastatic) indicating no differences of BACH1 levels between groups.

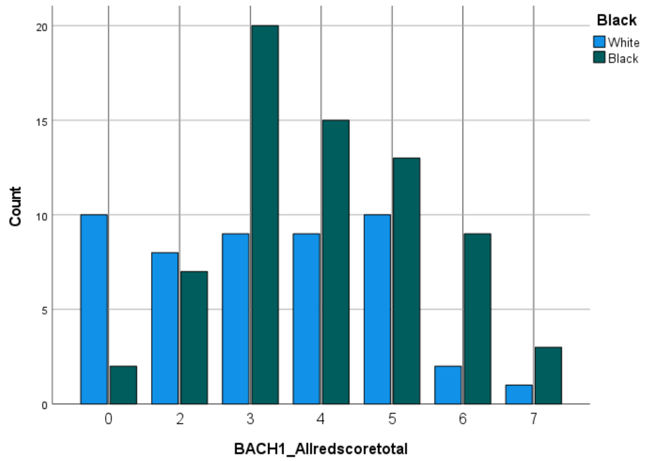

Supplementary Data Figure S4. BACH1 IHC score distribution in Black or White women. BACH1 IHC scores per patient are counted and plotted by race/ethnic group.

N.M.Dowling et al.
Supplementary Data Figure S5

A

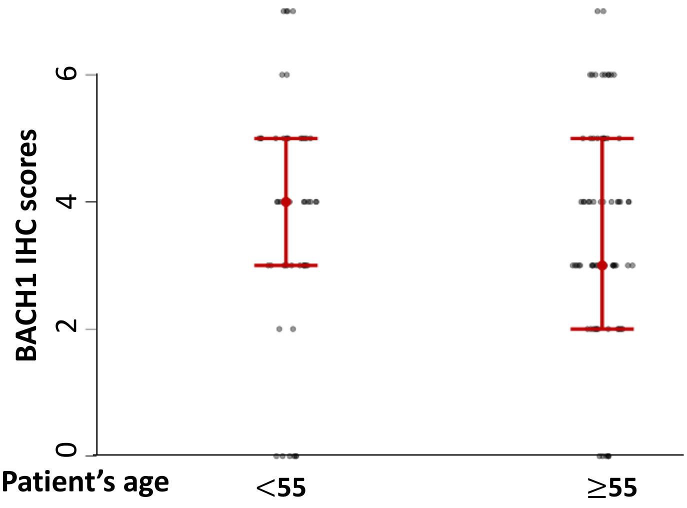

	Group	N	Mean	SD	SE	Coefficient of variation
**BACH1 scores**	< 55 yrs old	50	3.780	1.866	1.264	0.494
	>55 yrs old	73	3.521	1.725	0.202	0.490

B

	Age Group	N	Missing	Mean	SD	Min	Max
**BACH1 (Allredscoretotal)**	24-49	38	4	3.895	1.842	0	7
	50-65	42	4	3.429	1.863	0	7
	66-96	43	4	3.581	1.651	0	7

Factor	Kruskal-Wallis Test	df	*p*
**Age groups**	1.980	2	0.372

Supplementary Data Figure S5. BACH1 IHC scores is not associated with patient's age
(A) BACH1 IHC scores comparison in young patients (<55 years old, N = 50) and old patients (> 55 years old, N = 73) using Mann-Whitney U test (p = 0.257). Score mean (red dot), standard deviation (SD), standard error (SE) and coefficient of variation are shown by the subtype groups. (B) Patients are grouped in 3 categories by their age at the diagnosis in clinic to correlate BACH1 IHC scores with patient's age. Patient numbers, score mean, and standard deviation (SD) with maximum and minimum by age groups are indicated in the table. Age groups show no statistical difference of BACH1 scores (p = 0.372) using the Kruskal-Wallis test.

N.M.Dowling et al.
Supplementary Data Figure S6

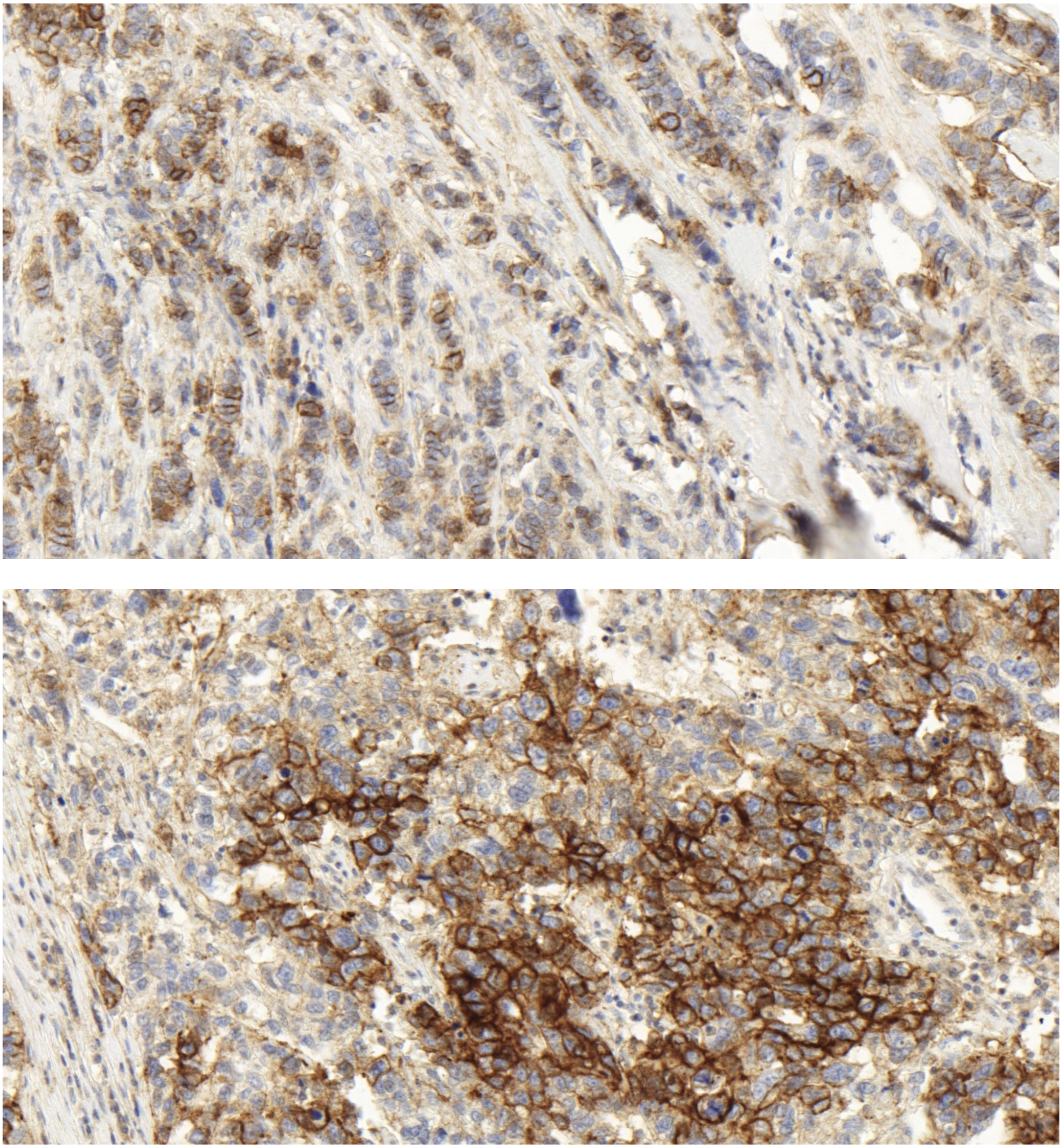

MCT1 (medium

MCT1 (high)

Supplementary Data Figure S6. Representative MCT1 IHC images
MCT1, a transporter protein, located on the cell membrane was stained by IHC in breast tumor tissues.

N.M.Dowling et al.
Supplementary Data Figure S7

A

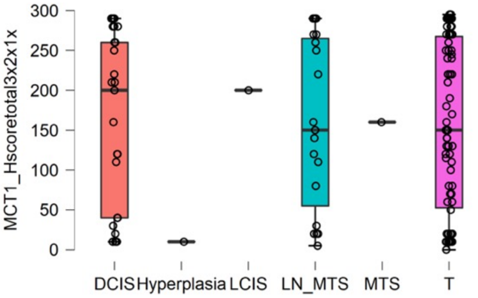

Tissue	DCIS	Hyperplasia	LCIS	LN_MTS	MTS	T
	MCT1_Hscoretotal3x2x1x					
N	25	1	1	19	1	78
Min	10	10	200	5	160	0
Max	290	10	200	290	160	295

B

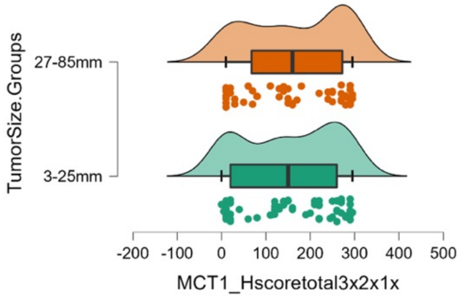

	Test	Statistic	df	p	Effect Size
MCT1	Student	-0.646	115	0.520	-0.120
	Mann-Whitney	1511.000		0.326	-0.106

C

	Subgroups	Number (N)	Mean Rank	Sum of Ranks
MCT1_Zscore	NON-INVASIVE (non-metastatic)	105	62.65	6578.50
	INVASIVE (metastatic)	20	64.83	1296.50
	Total	125		

Test Statistics (Mann-Whitney U and Wilcoxon W tests)	
	Zscore: MCT1_Hscoretotal(3x%+2x%+1x%)
Mann-Whitney U	1013.500
Wilcoxon W	6578.500
Z	-.246
Asymp. Sig. (2-tailed)	.805
Exact Sig. (2-tailed)	.808
Exact Sig. (1-tailed)	.404
Point Probability	.001
Grouping Variable: Invasive vs Non-Invasive Cancer	

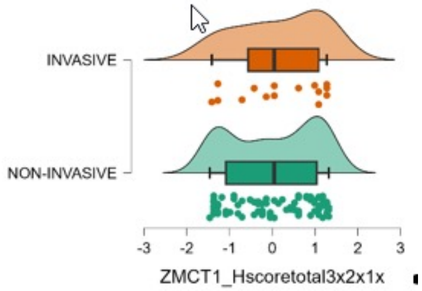

D

	Age Groups	N	Mean	SD
MCT1	24-49	41	178.171	104.599
	50-65	39	141.667	108.902
	66-96	44	148.182	102.646
**Factor**		Kruskal-Wallis Test	df	*p*
**Age Groups**		3.315	2	0.191

	Age	N	Mean	SD	SE	Coefficient
MCT1	below 55	52	169.90	103.524	14.356	0.609
	55 or older	72	146.04	106.708	12.576	0.731

Supplementary Data Figure S7. MCT1 expression levels are not associated with tumor tissue types, tumor size, patient's age, or marital status but higher in higher tumor grades

(A) Box plots indicating that MCT1 IHC scores are similar in different tissue types; DCIS, LCIS, LN-MTS, and tumors. Hyperplasia, LCIS and MTS tissue types are excluded for analyses due to its small sample size (N = 1). (**B**) Distribution plots indicating that MCT1 expression scores showed no difference in two groups by tumor size (small 3-25 mm vs. big 27-85 mm in diameter). For the statistical analysis, the effect size is given by Cohen's d for the Student's *t* test or by the rank biserial correlation for the Mann-Whitney U test. (**C**) Distribution plot of MCT1 IHC scores by subgroups (invasive vs. non-invasive) indicating no differences of BACH1 levels between groups. Mann-Whitney and Wilcoxon W test results are shown. (**D**) Patient age groups (Group I: 24-49 years old: N = 41, Group II: 50-65 years old: N = 39, Group III: 66-96 years old: N = 44) showed no statistical difference ($p = 0.191$) in MCT1 IHC scores by Kruskal-Wallis test. Patient age groups (Group I: below 55 years old: N = 52, Group II: above 55 years old: N = 72) showed no statistical difference ($p = 0.179$) in MCT1 expression scores by Mann-Whitney U test.
